# Predictive value of NoSAS questionnaire combined with the modified Mallampati grade for hypoxemia during routine sedation for gastrointestinal endoscopy

**DOI:** 10.1186/s12871-023-02075-3

**Published:** 2023-04-17

**Authors:** Nana Li, Junbei Wu, Yunhong Lu, Jigang Zhang, Zhaochu Sun, Xiaofei Cao, Cunming Liu

**Affiliations:** 1 Department of Anesthesiology, The First Affiliated Hospital of Nanjing Medical University, Nanjing, China; 2 Department of Anesthesiology, The Second Affiliated Hospital of Nanjing Medical University, Nanjing, China

**Keywords:** Hypoxaemia, Gastrointestinal endoscopy, NoSAS questionnaire, The modified Mallampati grade

## Abstract

**Background:**

The incidence of hypoxemia during painless gastrointestinal endoscopy remains a matter of concem. To date, there is no recognized simple method to predict hypoxemia in digestive endoscopic anesthesia. The NoSAS (neck circumference, obesity, snoring, age, sex) questionnaire, an objective and simple assessment scale used to assess obstructive sleep apnea (OSA), combined with the modified Mallampati grade (MMP), may have certain screening value. This combination may allow anesthesiologists to anticipate, manage, and consequently decrease the occurrence of hypoxemia.

**Methods:**

This study was a prospective observational trial. The primary endpoint was the incidence of hypoxaemia defined as pulse oxygen saturation (SpO2) < 95% for 10 s. A total of 2207 patients admitted to our hospital for painless gastrointestinal endoscopy were studied. All patients were measured for age, height, weight, body mass index, neck circumference, snoring, MMP, and other parameters. Patients were divided into hypoxemic and non-hypoxemic groups based on the SpO2. The ROC curve was plotted to evaluate the screening value of the NoSAS questionnaire separately and combined with MMP for hypoxemia. The total NoSAS score was evaluated at cut-off points of 8 and 9.

**Results:**

With a NoSAS score ≥ 8 as the critical value for analysis, the sensitivity for hypoxemia was 58.3%, the specificity was 88.4%, and the area under the ROC was 0.734 (*P* < 0.001, 95% CI: 0.708–0.759). With a NoSAS score ≥ 9 as a critical value, the sensitivity for hypoxemia was 36.50%, the specificity rose to 96.16%, and the area under the ROC was 0.663 (*P* < 0.001, 95% CI: 0.639–0.688). With the NoSAS Score combined with MMP for analysis, the sensitivity was 78.4%, the specificity was 84%, and the area under the ROC was 0.859 (*P* < 0.001, 95%CI:0.834–0.883).

**Conclusions:**

As a new screening tool, the NoSAS questionnaire is simple, convenient, and useful for screening hypoxemia. This questionnaire, when paired withMMP, is likely to be helpful for the screening of hypoxemia.

## Introduction

The purpose of gastrointestinal endoscopy is to achieve a thorough examination of the gastrointestinal tract in the safest and most comfortable manner possible. Although drug-induced sedation in endoscopic procedures improves patient comfort and facilitates endoscopic performance [1, 2], airway management complications during anesthesia are not rare [3, 4]. Therefore, hypoxemia related to airway management remains a matter of concern because of its potential impact on end-organ function and long-term outcomes [5]. Severe hypoxemia leads to anaerobic metabolism and circulatory changes, contributing to ischemia, especially in patients with coexisting diseases who are at greater risk of hypoxemic cardiac or cerebral damage [6]. Transient hypoxemia is thought to be a marker of increased risk of cardiovascular complications [7]. In fact, some risk factors can be screened and detected before procedures, allowing anesthesiologists to anticipate, manage, and consequently decrease the occurrence of hypoxemia. Therefore, the identification of patients at risk for hypoxemia is very important.

Previous studies have reported the incidence of hypoxemia during digestive endoscopy to range from 5.3 to 50% [7, 8].The profound suppression of upper airway muscle tone during deep sedation might account for the greater incidence of hypoxemia during sedation as compared to during normal sleep [9]. One study confirmed that patients with hypoxemia under sedation are likely to experience sleep apnea [10].

Unfortunately, there is no simple method for predicting hypoxemia during digestive endoscopic anesthesia. Instead, the method of assessing difficult airways in the operating room has been adopted, with other potentially relevant factors such as the position of the patient, endoscopic operation, bite placement, and other factors being ignored. Nonetheless, a single test that provides a simple, precise, and dependable prediction of hypoxemia is needed.

According to our preliminary experiment, patients who developed hypoxemia had a higher BMI and larger neck circumference. Interestingly, these two physical indicators are thought to correlate with obstructive sleep apnea (OSA) severity [11]. Under sedation, patients with hypoxemia are likely to have OSA. This led us to ask whether OSA assessment scales could be used to screen patients for hypoxemia? It was not logistically feasible to perform a sleep study on all patients undergoing endoscopy; therefore, we identified patients as having a high or low risk for sleep apnea based on a validated questionnaire, the neck circumference, obesity, snoring, age, sex (NoSAS) score. The NoSAS score is a new initial tool that can be used as a simple and easy measure of OSA [12].

A single clinical indicator, especially a highly subjective one, may be more accurate when combined with other, more objective indices. The modified Mallampati grade (MMP) is generally used to describe the relative relationship between the body of the tongue and the soft palate. Clinically, it is often used to identify difficult airways and still holds significant value among new predictors in the assessment of difficult laryngoscopy [13]. Studies have shown that MMP is a useful component of clinical examination that has clinical value in predicting the severity of OSA [14, 15].

However, whether it can be used to diagnose and evaluate hypoxemia remains unclear. Therefore, we aimed to evaluate the value of the NoSAS questionnaire combined with MMP in screening for hypoxemia during routine sedation for gastrointestinal endoscopy.

## Materials and methods

Our study population was selected from among a prospective cohort of patients who underwent gastrointestinal endoscopy at the First Affiliated Hospital of Nanjinng Medical University. Patients who underwent these procedures between May 2020 and November 2020 were enrolled in the study. The study was approved by the hospital ethics committee (Clinical trial: ChiCTR2000032801, first registered 11/05/2020). Patients were classified as hypoxemic (384 cases) or non-hypoxemic (1823 cases) based on oxygen saturation below 95%.

The included patients were ≥ 18 years old, had American Society of Anesthesiologists (ASA) physical status classification scores of I-III, and were scheduled for gastrointestinal endoscopy. Patients scheduled for endoscopic retrograde cholangiopancreatography and procedures performed under planned tracheal intubation, pregnant women, and emergency patients were excluded from the study.

**Fig. 1:**
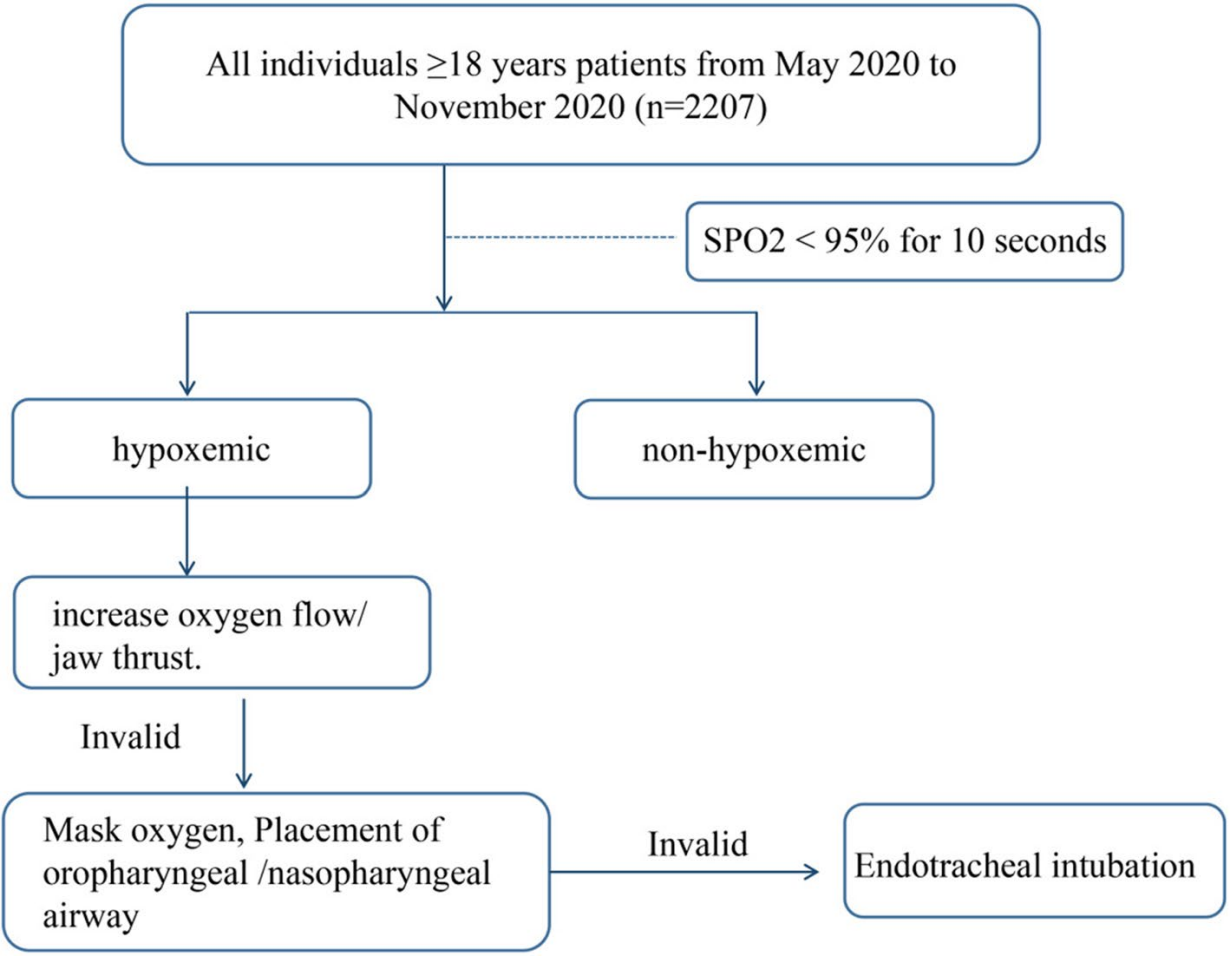
A fowchart elucidating the grouping methods included in this study, and management procedures for patients with hypoxemia

All patients remained in the left lateral position with their knees bent after admission and were monitored using continuous electrocardiography, heart rate, pulse oximetry, and noninvasive blood pressure monitoring during the procedure. Supplemental oxygen using double nasal catheters (5–7 L/min, lasting for 3 min) was provided to all patients. A trained professional was responsible for the maintenance of sedation and patient monitoring while using propofol. The induction dose of propofol was 1–2 mg/kg (obese patients were administered an induction dose based on ideal body weight). Upon loss of patient responses to verbal prompting, disappearance of the eyelash reflex, and absence of body motion response after firm pressing of the back edge of the mandibular branch, the digestive endoscopist was cleared to begin the operation. Propofol was added intraoperatively according to the patient's pain response, with supplementary doses consisting of 10–20 mg. In the case of SPO2 < 95% lasting more than 10 s, the anesthesiologists provider performed the necessary airway maneuver, which involved increase oxygen flow by opening the patient’s mouth. If the above measures proved ineffective, the operation was ceased, place the nasopharyngeal or oropharyngeal airway, or oxygen inhalation through the mask, and assist breathing (Fig. 1).

1. The recorded patient variables included sex, age, ASA physical status, height, weight, neck length, history of snoring, and MMP.
2. NoSAS questionnaire: All patients were instructed to complete the questionnaires provided to them by their anesthesiologist prior to examination. The questionnaire included the following items, along with the associated score values: neck circumference > 40 cm, 4 points; BMI between 25 and 30, 3 points; BMI ≥ 30, 5 points; snoring, 2 points; age > 55, 4 points; male sex, 2 points. A a threshold of 8 points or more indicates a high risk of OSA (Table 1) [12].
3. The MMP consisted of four classes: Class I, everything visible (tonsillar pillars); Class II, fully visible uvula; Class III, only the soft palate and base of the uvula visible; and Class IV, the soft palate not visible. MMP is a comprehensive assessment; the higher the grade, the more severe the airway stenosis.

**Table 1:** The NoSAS questionnaire

Indexes	Scores
Neck circumference > 40 cm	4
Obesity	
25 < BMI < 30 kg/m^2^	3
BMI ≥ 30 kg/m^2^	5
Snoring	2
> 55 yrs	4
Male	2

## Statistical analysis

All data were analyzed using SPSS version 25.0. For quantitative data, the independent Student’s t test was used for normally distributed data, and the Mann–Whitney test was used for non-normally distributed data. Qualitative data were evaluated using the χ2 test; Fisher’s exact probability method was used for variables that did not meet the χ2 test conditions. A multiple logistic regression method was used to create a multi-index joint diagnostic model. Hypoxemia as the cause quantity, continuous variable NoSAS rating, and MMP were used as variables, and the predictive value of the model was determined according to the regression equation. A receiver operator characteristic (ROC) curve was plotted to evaluate the screening value of the NoSAS questionnaire alone versus the MMP combined with the NoSAS questionnaire. Adjusted odds ratios and 95% confidence intervals (CIs) were calculated. All tests were two-sided at a significance level of 0.05.

## Results

### Baseline characteristics

Overall, 2207 patients, including 1136 men and 1071 women, were enrolled in this study. Of these, 384 (17.4%) experienced hypoxemia. The median age was 55 years (44-63 years) in the non-hypoxemic group and 57 years (48-65 years) in the hypoxemic group. According to the NoSAS questionnaire, the proportion of patients with NoSAS ≥ 8 was 11.6% and 58.3% in the non-hypoxemic and hypoxemic groups, respectively. According to MMP, 1644 patients were grade 1, 497 patients were grade 2, and 66 patients were grade 3. There was no difference in sex or ASA classification between the two groups (Table 2). Patients in the hypoxemic group had a higher average age, MMP, and neck circumference.

**Table 2:** Patient characteristics and preoperative risk factors for hypoxaemia (SpO2 < 95%) using univariate analysis

Variables	Non-hypoxaemia group (*n* = 1823)	Hypoxaemia group (*n* = 384)	X^2^/Z	*P*-Value
Age(yrs)	55 (44 ~ 63)	57 (48 ~ 65)	-3.466	0.001
BMI(kg/m^2^)	22.49 (20.57 ~ 24.45)	25.35 (23.42 ~ 27.74)	-15.792	< 0.001
Abdominal girth(cm)	78 (73 ~ 83)	87 (80 ~ 93)	-15.894	< 0.001
ASA physical status			-1.621	0.071
I	301 (16.5%)	57 (14.84%)		
II	1475 (80.9%)	308 (80.20%)		
≥ III	47 (2.6%)	19 (4.96%)		
The modified Mallampati grade (MMP)			-25.202	< 0.001
I	1551 (85.1%)	93 (24.2%)		
II	254 (13.9%)	243 (63.3%)		
≥ III	18 (1%)	48 (12.5%)		
Propofol dose (ug/kg/min)	181(152 ~ 233)	185(151 ~ 242)	-0.865	0.387
NoSAS related indexes				
Male (n,%)	925 (50.7%)	211 (54.9%)	2.248	0.134
Age(yrs)			5.989	0.014
≤ 55	956 (52.40%)	175 (45.60%)		
> 55	867 (47.60%)	209 (54.4%)		
Bullnecked (Neck circumference > 40cm)	124 (6.8%)	216 (56.3%)	595.107	< 0.001
Snoring	195 (10.7%)	199 (51.80%)	365.818	< 0.001
BMI(kg/m^2^)			-13.866	< 0.001
< 25	1469 (80.6%)	184 (47.9%)		
25 ~ 30	327 (17.9%)	160 (41.7%)		
≥ 30	27 (1.5%)	40 (10.4%)		
NoSAS scores	4 (2 ~ 6)	9 (4 ~ 11)	-16.904	< 0.001
NoSAS ≥ 8	212 (11.6%)	224 (58.3%)	436.442	< 0.001

**Fig. 2:**
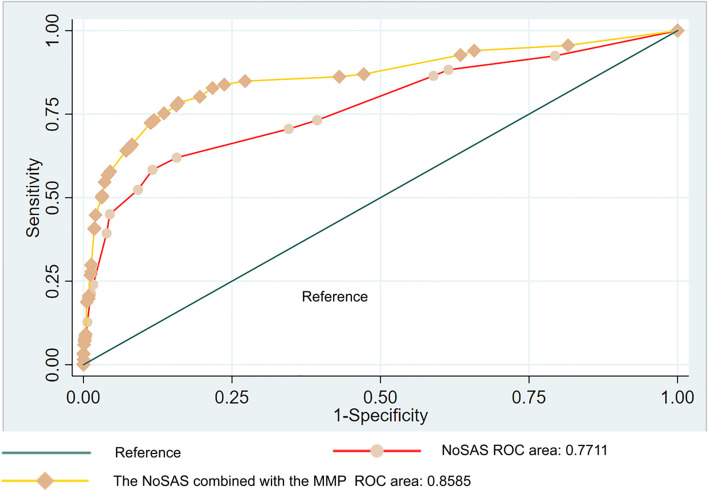
The ROC curve of the NoSAS questionnaire combined with the MMP for hypoxaemia

With a NoSAS score of 8 as a cutoff for analysis, the sensitivity and specificity for hypoxemia screening were 58.3% and 88.4%, respectively, and the area under the ROC curve was 0.734 (95% CI, 0.708–0.759) (Table 4). With a NoSAS score cutoff of 9, the sensitivity for hypoxemia dropped to 36.50%, but the specificity rose to 96.16%, with the area under the curve being 0.663 (*P* < 0.001, 95% CI:0.639–0.688) (Table 4). A multiple logistic regression method was used to create a multi-indicator joint diagnostic model (Table 3). The sensitivity and specificity of the NoSAS questionnaire combined with MMP for OSA screening were 78.4% and 84%, respectively, and the AUC was 0.859 (95%CI, 0.834–0.883) (Table 4 and Fig. 2). The results showed that, compared with the single NoSAS questionnaire, the sensitivity and AUC of the combined model were significantly improved. However, the NoSAS questionnaire with a score cutoff of 9 was still able to more precisely identify high risk patients.

**Table 3:** The NoSAS questionnaire combined with the MMP for hypoxaemia Logistics regression model

Variate	B	S.E	Wald	Sig
NoSAS	0.2310	0.0189	149.6919	< 0.001
The MMP	2.0631	0.1275	261.6277	< 0.001
Constant	-5.9437	0.2372	628.0094	< 0.001

**Table 4:** The value of NoSAS questionnaire combined with the MMP in hypoxaemia screening

	AUC	95% CI	S.E	Sensitivity	Specificity	Sig
NoSAS ≥ 8	0.734	(0.708 ~ 0.759)	0.013	58.3%	88.4%	< 0.001
NoSAS ≥ 9	0.663	(0.639 ~ 0.688)	0.013	36.5%	96.16%	< 0.001
NoSAS combined with the MMP model	0.859	(0.834 ~ 0.883)	0.013	78.4%	84%	< 0.001

## Discussion

Major complications of airway management are still a matter of concern, and hypoxemia remains the most common cause of death during anesthesia [7]. Non-tracheal anesthesia under sedation presents a further challenge to anesthesiologists. Thus, proper assessment of the airway is important to prevent hypoxemia. Since gastroenteroscopic surgery is fast paced, simple clinical characteristics that can predict hypoxemia and allow for expedited assessment is highly attractive.

Previous studies have confirmed that patients with oxygen desaturation under conscious sedation may be considered for sleep apnea screening [10]. Our preliminary study also found that patients with body shapes similar to those of suspected OSA patients (e.g. obesity, bullnecked, snoring) were more likely to develop hypoxemia under sedation. Compared to the non-hypoxemic group, hypoxemic patients were more likely to be obese (*P* < 0.05). The frequency of MMP > 2 was higher, and more than 50% of the patients had a history of snoring (*P* < 0.05). It was not logistically feasible to perform a sleep study on all patients undergoing endoscopy, so we identified patients as having a high or low risk for sleep apnea based on the NoSAS questionnaire to explore whether people at high risk for sleep apnea were more likely to develop hypoxemia.

At present, many scales are used in clinical screening for OSA, such as the STOP-BANG questionnaire, Berlin questionnaire, and NoSAS score. Indeed, Harvin et al. found that patients with a high risk of sleep apnea, as assessed using the Berlin score, had a higher rate of hypoxemia during conscious sedation for colonoscopy [16].

However, the first two items included a variety of subjective factors, such as snoring, fatigue, and drowsiness. These indicators are easily influenced by the ability to understand, cognition, and education of patients. The NoSAS score is determined by less subjective metrics, such as age, sex, neck circumference, and BMI, with the only subjective item being snoring (its retention being due to its strong statistical association with OSA) [12]. The scale is objective and easy to operate. It has been documented that the NoSAS questionnaire effectively aids clinicians in quickly addressing nocturnal hypoxia in patients with cerebral infarction [17]. Using a threshold of 8 points or more, the NoSAS score identified individuals at a high risk of clinical OSA. We found that individuals with high NoSAS scores were more likely to develop hypoxemia. Patients in the hypoxemic group had a higher BMI and a larger proportion of bullneckedness, with these two indicators accounting for a larger proportion of patients with NoSAS. Although using a cutoff value of ≥ 8 for the NoSAS questionnaire had better sensitivity and greater area under the ROC curve than a cutoff of ≥ 9 in predicting hypoxemia, it had lower specificity. Therefore, we judge a NoSAS score of 9 to be more helpful in screening patients with hypoxemia. The deleterious impact of obesity on respiration under anesthesia are well appreciated. Because of excess weight on the chest wall and diaphragm, the lung compliance, functional residual capacity, functional vital capacity, total lung volume, total lung capacity, and expiratory reserve volume are all reduced in obese patients [18, 19]. In other words, obesity itself is more likely to lead to hypoxemia. Moreover, OSA is not the only mechanism leading to hypoxemia during sedation and anesthesia, and obese patients are especially susceptible to physiological changes which can negatively affect pulmonary function during anesthesia. Patients with high NoSAS scores (≥ 8) were more likely to develop hypoxemia, and their sensitivity was higher than that of the OSA screening. A possible reason is that the propensity for the upper airway to collapse increases with increasing depth of propofol anesthesia [20]. In our study, the total amount of propofol was higher in the hypoxemic group than in the non-hypoxemic group, but there was no difference after controlling for weight and duration of surgery (median was 185 vs. 181). The induction dose of propofol was 1–2 mg/kg (obese patients were administered an induction dose based on ideal body weight). The infusion rate was slow, and the depth of anesthesia was judged by an experienced anesthesiologist. The occurrence of hypoxemia in patients does not always occur after induction but may instead occur throughout the operation. Therefore, we did not compare the induction dose of propofol but rather the total amount. Despite this, we believe that patients with high NoSAS scores are more likely to develop hypoxemia under sedation.

The MMP is thought to be a simple and quick instrument for assessing airway patency before intubation [21]. Previous studies have pointed out the association between a higher MMP and the severity of OSA [15]. However, the current opinion is that MMP is no longer useful as a standalone predictor; in combination with others, though, it is still part of today's most relevant guidelines. To determine the extent of pharyngeal stenosis, MMP can be used to diagnose and evaluate hypoxemia under sedation. We found that the combination of MMP with the NoSAS questionnaire yielded a slightly reduced specificity, but a significantly increased sensitivity and area under the curve in the combined model.

The advantage of this study is that it represents one of the most recent clinical studies to analyze the applied value of the NoSAS score combined with MMP in diagnosing hypoxemia during routine sedation for gastrointestinal endoscopy. These indicators are relatively objective, concise, and suitable for fast-paced ambulatory surgeries. However, this study is, of course, not without its limitations. First, our study was conducted in only one single center. These results require further validation in polycentric and multiracial populations. The NoSAS questionnaire is based on a European population, and there may be ethnic differences in its screening effectiveness. Second, the patients included in this study only had ASA I-III; these findings may not hold true in ASA IV-V patients who are at a much higher risk of developing hypoxemia and other adverse events. Third, we did not assess the independent predictive effect of MMP on hypoxemia. Although in this study, the evaluation of the MMP was carried out in a unified position and strictly in accordance with the study’s standards, such research results may still not be universally applicable, as this is a single indicator and may lead to different grades due to the different patient positions. Poor inter-examiner agreement may have affected their predictive value. In summary, the NoSAS questionnaire combined with MMP can significantly improve the sensitivity and specificity of hypoxemia, which can be used clinically for hypoxemia screening to facilitate early detection and intervention.

## Conclusions

As a new screening tool, the NoSAS questionnaire is simple, convenient, and has a certain screening value for hypoxemia, especially with the NoSAS score cutoff of 9. Combined with the modified Mallampati grade (MMP) may be more helpful to the screening of hypoxemia.

## Data Availability

The datasets used and/or analysed during the current study available from the corresponding author on reasonable request.

## Abbreviations

MMP: The modified Mallampati grade
OSA: Obstructive sleep apnea
SPO2: Pulse oxygen saturation
AUC: Area under curve
ROC: Receiver operating characteristic curve
BMI: Basal metabolic rate
ASA: American Society of Anesthesiologists
